# An Evaluation of Risk Attitudes and Risk Tolerance in Emergency Medicine Residents

**DOI:** 10.7759/cureus.4451

**Published:** 2019-04-13

**Authors:** Carlos Rodriguez, Nishad A Rahman, Kory London, Robin Naples, Simran Buttar, Xiao Chi Zhang, Hyunjoo Lee, Joshua Rudner, Dimitrios Papanagnou

**Affiliations:** 1 Emergency Medicine, Thomas Jefferson University, Philadelphia, USA; 2 Emergency Medicine, Sidney Kimmel Medical College, Thomas Jefferson University, Philadelphia, USA; 3 Emergency Medicine, Maimonides Medical Center, Brooklyn, USA; 4 Emergency Medicine, Stony Brook University Hospital, Stony Brook, USA

**Keywords:** risk, risk tolerance, wellness, risk attitude, risk type, emergency medicine, graduate medical education

## Abstract

Introduction

Previous studies have shown that risk attitudes and tolerance for uncertainty are significant factors in clinical decision-making, particularly in the practice of defensive medicine. These attributes have also been linked with rates of physician burnout. To date, the risk profile of emergency medicine (EM) physicians has not yet been described. Our goal was to examine the risk profile of EM residents using a widely available risk tolerance and attitude assessment tool.

Methods

First-, second-, and third-year residents of Thomas Jefferson University Hospital’s EM residency program completed the commercially available, unmodified Risk Type Compass, a validated instrument offered by Multi-Health Systems (MHS Inc, New York, USA). Scored reports included information on residents’ risk type (one of eight personality types that reflect their temperament and disposition); risk attitudes (domains where residents are more likely to engage in risky behaviors); and an overall risk tolerance indicator (RTi) (a numerical estimate of risk tolerance). RTi scores are reported as means with 95% confidence intervals (CIs).

Results

There was no significant change in RTi scores in residents across different years of their post-graduate year (PGY) training. PGY-one residents trended towards risk aversion; PGY-two residents were more risk-taking; and PGY-three residents scored in the middle.

Conclusion

Our pilot assessment of risk types in EM residents highlighted shifts across the years of training. Variations between members of each PGY cohort outweighed any outright differences between classes with regards to absolute risk tolerance. There was an increase in the frequency of health and safety risk-taking attitude with higher PGY class, and this was also the risk attitude that was the prominent domain for resident risk tolerance. The study was limited by sample size and single cross-sectional evaluation.

## Introduction

Despite significant advances, healthcare remains a field steeped in uncertainty and ambiguity. There are intrinsic risks whenever any management plan is executed. These risks pose even graver challenges in emergency medicine (EM), where uncertainty intersects with patient acuity and patient volume.

While high-risk decisions and management are replete in the practice of EM, the risk profiles of EM providers, particularly those in training, have not been assessed by prior studies. Knowledge of risk-taking tendencies may be critical in advising and developing physicians in training. EM providers with substantial risk aversion may subject their patients to unnecessary testing, such as advanced diagnostic imaging [1]. These providers may also be more likely to admit their patients or highly utilize observation units [2]. In addition, cognitive biases and personality traits (i.e., omission bias, tolerance to risk, and/or overconfidence) may lead to diagnostic inaccuracies and medical errors resulting in mismanagement or inadequate resource utilization [3].

It is imperative that providers reflect on their individual risk tolerances. Given there is no clear understanding of what ideal risk aversion or risk tolerance should be in EM residents, it is prudent to first identify a baseline risk profile for this group. While several studies have identified methodologies for assessing risk-taking behaviors and attitudes, they are not specific to EM physicians. To date, previous studies in this area have not utilized standardized assessments; consequently, cross-study comparisons still remain a difficult challenge.

The solution may be found by seeking guidance from other disciplines. Studies have demonstrated improved attitudes toward team building, communication, and adverse event recognition after having clinical teams from various disciplines (i.e., EM, surgery, nursing) complete coursework in crew resource management (CRM) [4-6]. CRM can easily be applied to EM providers, as they naturally work in teams immersed in high-stakes situations while managing fatigue, communication, and decision-making. Risk tolerance has been heavily studied in business, where risk attitudes are essential to employee recruitment, coaching, and strategic planning. Risk attitudes and their subsequent effects on entrepreneurship [7-8], small businesses [9], investments [9], and role conflict [10] have also been studied.

Using a validated risk assessment tool can be of potential benefit when assessing professionals across various fields and specializations. The Risk Type Compass (RTC), created in 2011 by the Psychological Consultancy Ltd (Tunbridge Wells, United Kingdom), provides multiple modes of risk assessment [11]. Our pilot study aimed to describe the risk type and risk tolerance of EM residents in our three post-graduate year (PGY) cohorts. We hypothesized that EM residents would tend to have a higher risk tolerance when compared to the general population, in line with existing ‘cowgirl’ and ‘cowboy’ stereotypes. Additionally, we hypothesized that risk tolerance would increase with the increasing PGY.

## Materials and methods

Study instrument

The RTC is a commercially available psychometrically validated assessment tool (Multi-Health Systems, New York, USA, 2011). The standard RTC questionnaire, which consists of two parts, is a 102-item assessment: 72 items that determine risk type, 20 items that determine risk attitude, and 10 items for validity. It includes a self-report with a six-point Likert scale for part one, and a three-point rank ordering for part two. For this pilot, the complete version of the questionnaire was used with official individualized scoring from RTC through Multi-Health Systems (MHS) in the form of a personal report. The RTC uses a normative sample of 7,072 adults (2015 sample). This particular evaluation has proven to have notable internal consistency, with α values greater than 0.8. Total RTC scores were computed by MHS software. For this pilot study, we used the RTC-generated personal report, which is not professional- or industry-specific.

The RTC offers several measures. ‘Risk type’, analogous to the eight personality types, is one such measure. Risk types include: wary (i.e., self-disciplined and cautious), prudent (i.e., self-controlled and detailed), deliberate (i.e., systematic and compliant), composed (i.e., cool-headed and optimistic), adventurous (i.e., impulsive and fearless), carefree (i.e., spontaneous and unconventional), excitable (i.e., uninhibited and excitable), and intense (i.e., pessimistic and self-critical). Risk type correlates to a spectrum for one’s relationship with risk, from risk-averse (i.e., wary) to risk-tolerant (i.e., adventurous).

The second measure is the ‘risk attitude’, which includes five sub-scales of risk tolerance across the different domains of one’s life. Risk attitudes include: financial (i.e., willingness to take financial risk), social (i.e., risk of embarrassing oneself or others and risking disapproval), health and safety (i.e., being alert to dangers that may impact one’s current or future health state), recreational (i.e., possibility of physical danger and its influence on decisions regarding recreational activities one engages in), and reputational (i.e., morality and a readiness to live life according to accepted principles).

Finally, the Risk Tolerance Index (RTi), an overall numerical estimate of tolerance for risk based on risk type and risk attitude, is calculated by combining the two measures. RTi ranges from zero (low risk tolerance) to 100 (high risk tolerance).

Study design and participants

This cross-sectional investigation was conducted at an urban EM residency training program. Study recruitment included 38 EM residents (13 PGY-one, 13 PGY-two, and 12 PGY-three residents) at a single academic medical institution, Thomas Jefferson University Hospital in Philadelphia, Pennsylvania. There were no exclusion criteria for participation. The RTC was deployed electronically in August 2017. Residents completed an electronic consent form followed by a demographics questionnaire including age, gender, and level of residency training.

RTi scores are reported as means with 95% CIs. Differences in risk tolerance based on PGY-training level were assessed. An analysis of variance (ANOVA) was used to evaluate differences across means. Study investigator DP has had exposure to MHS products through graduate-level doctoral studies, and primarily assisted with RTC score interpretation. This study was previously presented as a poster at a local symposium (Poster: Rahman N, Papanagnou D. An evaluation of risk attitudes and risk tolerance in emergency medicine. College within the College (CwiC) posters; November 20, 2017). The study was reviewed and received approval from the institutional review board of Thomas Jefferson University (#16E.646). 

## Results

**Figure 1:**
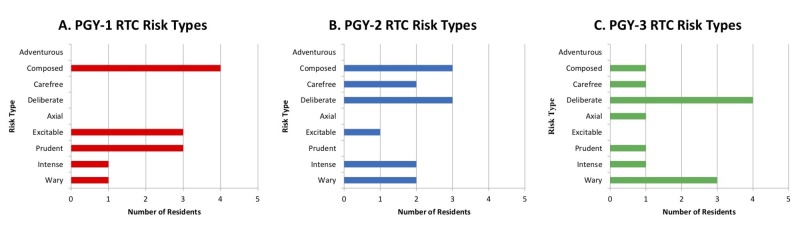
Risk Type Compass: Risk Types of (A) PGY-one, (B) PGY-two, and (C) PGY-three Residents. RTC: Risk Type Compass PGY: Post-graduate year

PGY-one EM residents gravitated towards the risk averse part of the spectrum. PGY-two and PGY-three residents were noted to cluster at both (i.e., risk-averse and risk-tolerant) ends of the risk-type spectrum (Figure 1).

**Figure 2:**
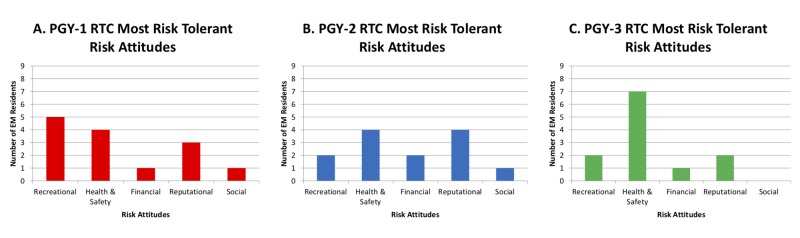
Risk Type Compass: Risk Attitudes of PGY-one (A), PGY-two (B), and PGY-three (C) Residents. PGY: Post-graduate year EM: Emergency medicine RTC: Risk Type Compass

When it comes to risk attitudes, PGY-one residents were most risk tolerant with regards to the recreational aspects of their lives. PGY-two residents were most risk tolerant with regards to their own health and safety, as well as their reputation. PGY-three residents were most risk tolerant when it came to their own health and safety (Figure 2)

RTi scores from the RTC assessment were compared across PGY levels (Table 1). RTi scores decreased as year of training increased, suggesting decreasing risk tolerance over training. Statistically significant differences in RTi means, however, were not observed across PGY resident groups (one-way ANOVA: F-ratio=0.11, p=0.89). 

**Table 1:** Risk Tolerance Index (RTi) for Residents per Post-graduate Year. N: Total number RTi: Risk type index Std. Dev.: Standard deviation PGY: Post-graduate year

Total RTi Per PGY-Year				
PGY-Year	N	Mean	Std. Dev.	95% Confidence Interval
PGY-one	13	47.23	12.50	6.79 [40.44 - 54.02]
PGY-two	13	46.69	13.88	7.55 [39.14 - 54.24]
PGY-three	12	44.83	13.24	7.49 [31.59 - 58.39]

## Discussion

The aim of this article was to describe the risk type and risk tolerance of EM residents by using the Risk Type Compass, a commercially available risk tolerance assessment tool. The RTC tool specifically focused on differences in how individuals perceive, react to, and manage risk, as well as how they make decisions when risk is involved [11].

A significant difference in RTi scores across years in training was not observed. While there were noted preferences for risk type by PGY class in our cohort, however, RTi, as a measure for risk tolerance, cannot be used to fully appreciate the nuances in changes in risk type across residents. Examination of resident risk attitudes is useful for identifying in which domains residents were more comfortable with risk-taking behaviors. This was most distinctive amongst PGY-three residents, who were most risk tolerant when it came to their own health and safety. Furthermore, ‘health and safety’ was one of the most commonly delineated risk attitudes for the highest risk tolerance, with increasing frequency from PGY-one to PGY-three residents.

While definitive conclusions are limited in our study, the evaluation of one's relationship with risk and tolerance for uncertainty in the clinical setting has the potential to offer valuable insight into trainee performance. Previous studies have linked tolerance for uncertainty with burnout in both emergency medicine physicians and residents. Kuhn et al. reported that poor tolerance for uncertainty, secondary to concerns for bad outcomes, is strongly correlated with emotional exhaustion and/or burnout in EM physicians [12].

Another study by Takeyesu et al. found that residents with higher levels of burnout were significantly less tolerant of uncertainty when it came to clinical decision-making. The authors also reported that uncertainty may serve as a source of anxiety and psychological stress, which may ultimately negatively affect clinical performance and delay informational recall, which collectively can inhibit optimal learning [13].

In light of these findings, directly addressing areas of uncertainty during residency training may be helpful in supporting EM residents as they build their clinical skills and develop their resilience to protect them from burnout. Additional studies applying the RTC risk assessment may be of use, especially when applied to cohorts longitudinally to assess potential shifts in risk tolerance over the course of their residency training. Such findings would offer insight into interventions that can impact education and training; wellness and burnout; and clinical decision-making. A potential opportunity for further investigation would be to include an objective measure for burnout, such as the Physicians’ Reaction to Uncertainty (PRU) Scale [14], to draw conclusions about the relationship between risk tolerance and burnout in specific cohorts of trainees.

While our study suggests several trends, it was hindered by the small sample size of only 38 EM residents from a single institution. Subsequent follow-up studies to this pilot should be adequately powered to note any differences in risk patterns. The study also represents a single snapshot of residents over the course of their training. It is possible that randomness in recruitment may impact trends between classes, where findings may actually represent differences in individual personalities, rather than effects based on training level. Moving forward, our study should be replicated with more EM residents across multiple institutions and repeated over the course of their training, in order to build a more robust and actionable risk profile.

## Conclusions

Risk type and tolerance inevitably affect patient care in the EM setting. Further research is needed to determine the ideal risk profile that allows for reduction in wasteful expenditures and defensive medicine without negatively impacting patient care. This data could also help inform curricular training and identify residents who may benefit from targeted wellness interventions. Our pilot study supports the notion that risk type and risk tolerance are aspects of provider personality worthy of additional scholarship.
